# A Single‐Nucleotide Substitution Generates a *de Novo* Promoter That Activates a Latent Metabolic Bypass in *Escherichia coli*

**DOI:** 10.1111/mmi.70092

**Published:** 2026-06-26

**Authors:** Qiaoqiao Guo, Qi Zou, Jilong Qin, Makrina Totsika, Yaoqin Hong, John E. Cronan

**Affiliations:** ^1^ Department of Microbiology, School of Molecular and Cellular Biology University of Illinois Urbana Illinois USA; ^2^ State Key Laboratory of Tropical Oceanography, South China Sea Institute of Oceanology Chinese Academy of Sciences Guangzhou Guangdong China; ^3^ Laboratory of Tropical Marine Bio‐Resources and Ecology, South China Sea Institute of Oceanology Chinese Academy of Sciences Guangzhou Guangdong China; ^4^ Centre for Immunology and Infection Control, School of Biomedical Sciences Queensland University of Technology Brisbane Queensland Australia; ^5^ Biomedicine and Molecular Biology, College for Medicine and Dentistry James Cook University Townsville Queensland Australia; ^6^ Australian Institute of Tropical Health and Medicine James Cook University Townsville Queensland Australia; ^7^ Department of Biochemistry, School of Molecular and Cellular Biology University of Illinois Urbana Illinois USA

## Abstract

The *Escherichia coli* *fabH* gene encodes the 3‐ketoacyl‐ACP synthase III that initiates fatty acid synthesis. Deletion of the *fabH* gene results in severely limited fatty acid synthesis and tiny cells that are unusually sensitive to antibiotics. Genetic investigations identified the *yiiD* gene, now called *madA*, that encodes a malonyl‐ACP decarboxylase that suppresses the *∆fabH* phenotype, but only when *madA* is multicopy. We selected vancomycin‐resistant suppressor derivatives of a *∆fabH* strain. Although such chromosomal mutations were generally rare and weak, suppressor strain s1 (a single C‐T transition) restored both wild‐type growth, high‐level vancomycin resistance, and wild‐type fatty acid synthesis by creation of a new promoter within the coding sequence of a gene upstream of *madA*. This provides a caveat to the extensive effort to develop FabH inhibitors as antibacterial drugs.

## Graphical abstract

A chromosomal mutation allows bypass of deletion of the *Escherichia coli* *fabH* gene. The bypass results from creation of a new promoter that drives increased *madA* transcription, resulting in increased synthesis of acetyl‐acyl carrier protein by decarboxylation of malonyl‐acyl carrier protein. The acetyl‐acyl carrier protein acts as the primer for long chain fatty acid synthesis.

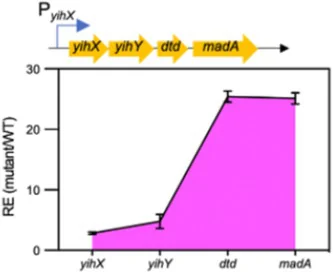

## 1 Introduction

Inhibition of a core metabolic pathway is widely assumed to impose a high barrier to evolutionary escape. This assumption underpins many antibacterial discovery strategies. However, this view often overlooks the possibility that latent compensatory activities may already exist within the cell but remain inaccessible due to regulatory constraints. Fatty acid biosynthesis provides a well‐studied example of this problem. In *E. coli* , FabH initiates fatty acid synthesis by condensing acetyl‐CoA with malonyl‐ACP to form 3‐ketobutyryl‐ACP (Tsay et al. 1992), which is converted to butyryl‐ACP which primes synthesis of the acyl chains of membrane phospholipids and lipid A (White et al. 2005; Cronan Jr. and Rock 2008).

The role of FabH in initiation argued that *fabH* was an essential gene in *E. coli* as was first reported (Lai and Cronan 2003). However, others found that Δ*fabH* strains remained viable, albeit slow growing and of tiny cell size (Baba et al. 2006; Yao et al. 2012). Clone‐bank complementation of a *∆fabH* strain identified the *yiiD* gene as a multicopy (p)ppGpp‐regulated suppressor of *fabH* null mutations (Sanyal et al. 2019), for which the inability to construct a ∆*yiiD* ∆*fabH* strain indicated synthetic lethality (Sanyal et al. 2019). The conflicting results in *fabH* essentiality were therefore due to different strain backgrounds. The original *E. coli ∆fabH* deletion was constructed in a strain deficient in synthesis of the stress “alarmone” nucleotides (p)ppGpp (guanosine tetraphosphate and pentaphosphate) whereas the viable ∆*fabH* strains were proficient in (p)ppGpp synthesis.

Later the *yiiD* encoded protein (renamed MadA) was shown to decarboxylate malonyl‐ACP to acetyl‐ACP that primed fatty acid synthesis (Whaley et al. 2021). Although the biochemical bypass was known, an unresolved important question was the requirement for multicopy expression of MadA for *∆fabH* bypass. In wild‐type strains, loss of FabH sensitizes *E. coli* to several otherwise ineffective antibiotics (Yao et al. 2012; Hong et al. 2022). Wild‐type *E. coli* cells are resistant to vancomycin because the intact outer membrane blocks access of the antibiotic to its peptidoglycan target, whereas *E. coli* *fabH* null mutants are susceptible due to a defective outer membrane (Yao et al. 2012; Hong et al. 2022). FabH has long been an antimicrobial target (documented by 130 PubMed entries). Hence, Δ*fabH* suppressors that restored growth and vancomycin resistance would provide a straightforward escape route from FabH inhibition.

A rare spontaneous vancomycin‐resistant Δ*fabH*‐suppressor, strain s1, containing a single cytosine‐to‐thymine (C → T) transition in the *yihY* gene upstream of *madA* created a highly active promoter for *madA* expression. Elevated MadA expression restored fatty acid synthesis, improved membrane function, and restored vancomycin resistance to the Δ*fabH* mutant. Thus, a single *cis*‐regulatory rewiring unlocked a latent metabolic bypass.

## 2 Results

### 2.1 A Suppressor Mutation That Restores Vancomycin Resistance to *E. coli* Δ*fabH* Strains

**FIGURE 1:**
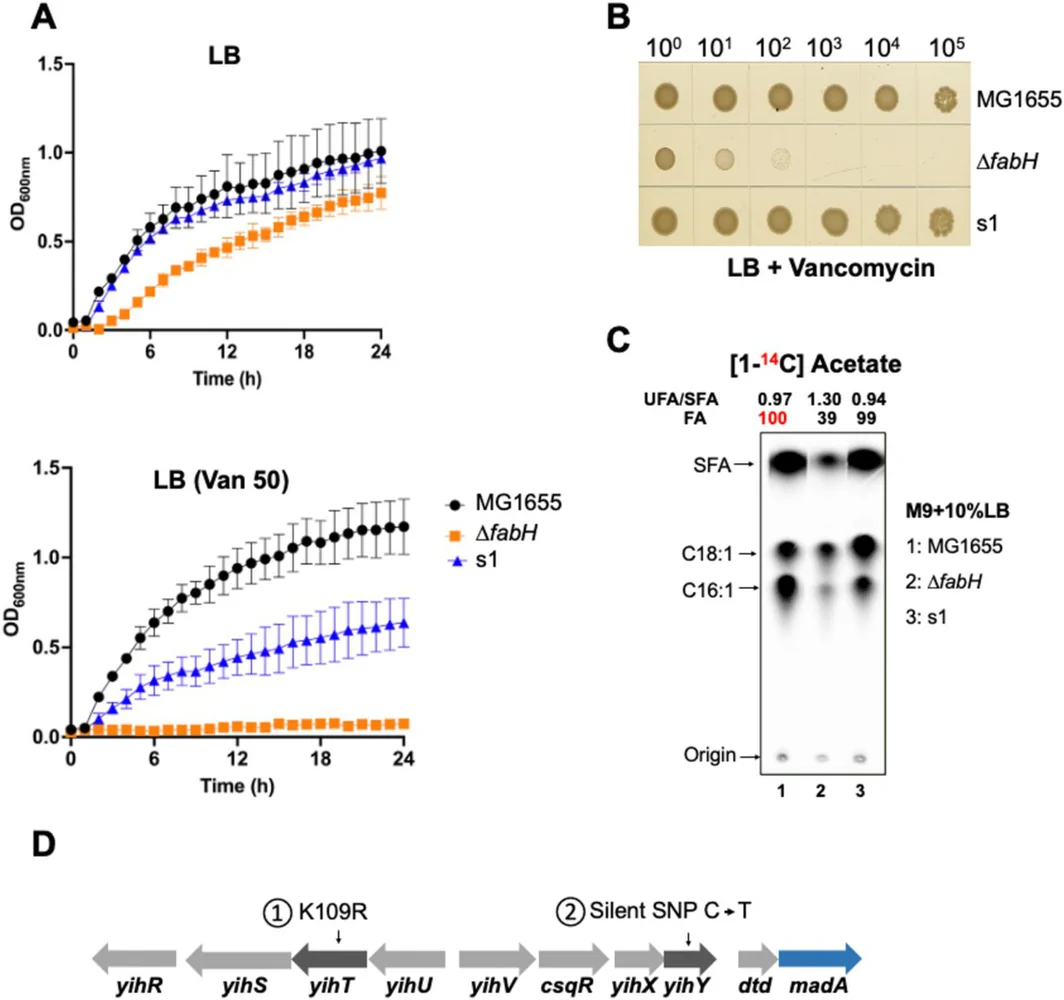
Identification of a suppressor isolate (s1) of MG1655 ∆*fabH* mutant. (A) Growth measurement of the *E. coli* ∆*fabH* strain and its s1 suppressor in LB medium with and without vancomycin. (B) The suppressor mutation of isolate s1 restored vancomycin resistance to the ∆*fabH* strain (the numbers above the figure panel indicate the dilution factor from OD_600_ 1.0 overnight cultures). (C) The s1 suppressor mutation reversed the defective *de novo* fatty acid synthesis in the ∆*fabH* strain. The numbers above the lanes showed the values for phospholipid acyl chain unsaturation and the radioactive label incorporation relative to the value (100) for the MG1655 strain. (D) Single‐nucleotide polymorphisms (SNPs) identified in suppressor isolate s1. The *yihX*, *yihY*, *dtd*, *madA* operon is reported to be transcribed from a promoter located immediately upstream of the *yihX* coding sequence (Mendoza‐Vargas et al. 2009). Note that the lines between the lanes of all TLC plates of this report were made prior to sample loading by scribing the coating of the plate with a rake‐like tool. This prevents bleeding of spots between lanes and simplifies quantitation.

The s1 suppressor was isolated by plating a culture of the *∆fabH* strain on LB agar plates containing 100 μg/mL vancomycin. The s1 suppressor strain restored wild‐type MG1655 growth rates in LB medium, reestablished essentially full vancomycin resistance and remedied the damaged cell membrane of ∆*fabH* strains (Figure 1A,B, Figure S2). Moreover, suppressor s1 restored the wild‐type level of fatty acid synthesis to the *∆fabH* strain (Figure 1C) shown by [1‐^14^C] acetate incorporation into membrane phospholipid acyl chains (Figure 1C). Whole‐genome sequencing of strain s1 showed two single‐nucleotide polymorphisms (SNPs), a thymine‐to‐cytosine (T → C) transition in the *yihT* gene and a C → T transition within the *yihY* gene, a silent mutation that retained the YihY residue 270 valine (Figure 1D). The vancomycin selection gave other suppressors, but these strains grew as poorly as the ∆*fabH* strain and were not further analyzed.

### 2.2 Suppression of the Δ*fabH* Null Mutation Is due to a Point Mutation Within the *yihY* Gene That Created a Promoter

**FIGURE 2:**
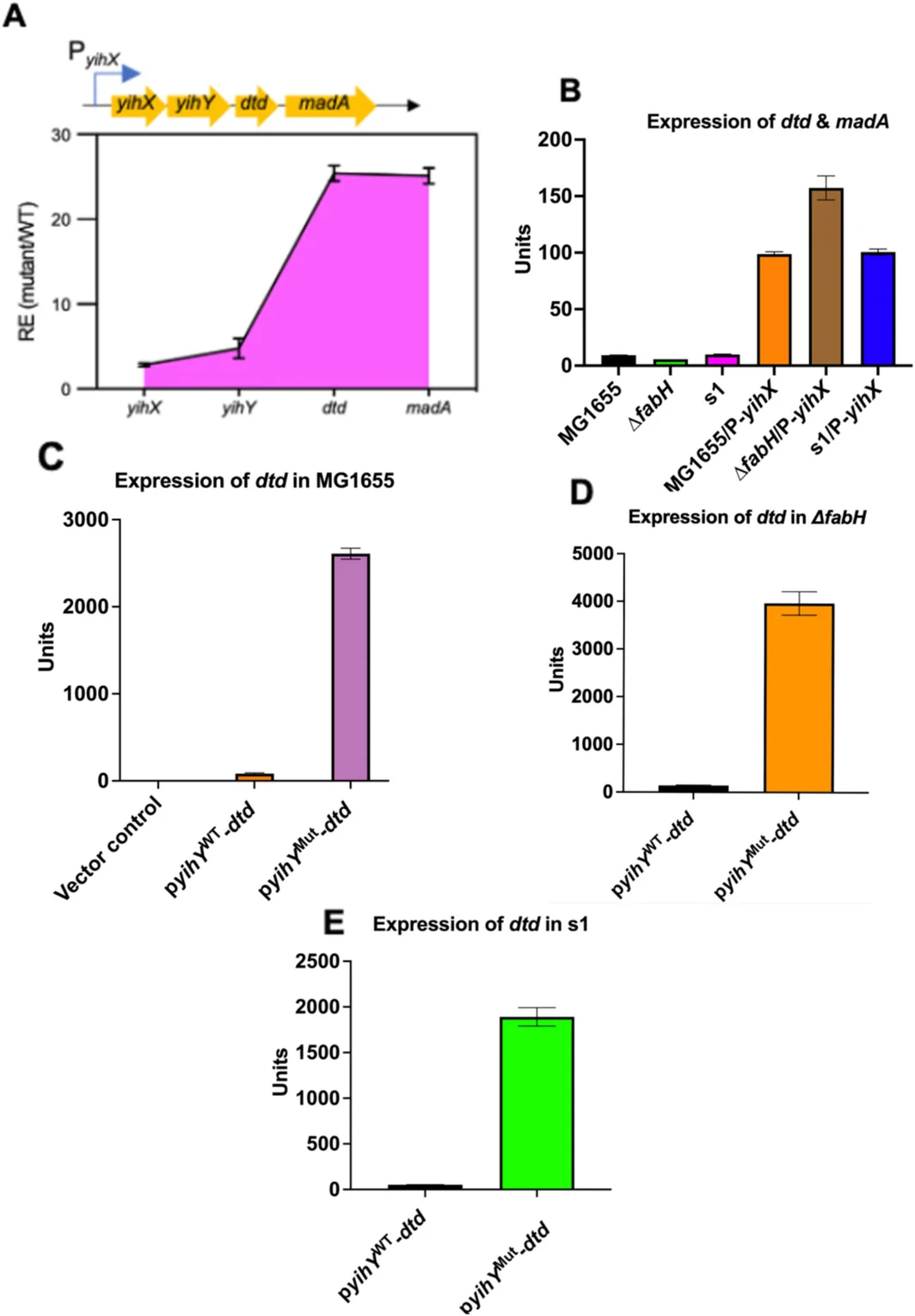
A single C → T transition mutation in *yihY* gives a *de novo* promoter that drives high‐level expression of the *dtd‐madA* region. (A) Transcriptomic analysis identified strong upregulation of *dtd* and *madA* in the suppressor isolate s1 compared to the parental ∆*fabH* strain. The *yihX–yihY–dtd–madA* operon structure is shown schematically. (B) A *lacZ* transcriptional fusion to the native *yihX* promoter showed that the *yihY* C → T mutation did not significantly alter transcription from the promoter upstream of *yihX in* the MG1655, ∆*fabH*, and s1 strains. LacZ transcriptional fusions containing the mutant *yihY* sequence confer a ~30‐fold increase in transcriptional activity of the downstream *dtd–madA* region in the three strains (C) strain MG1655, (D) strain Δ*fabH*, and (E) strain s1 whereas the wild‐type sequence had only basal activity. The fusions were constructed using the low copy vector, pHSG575 (Takeshita et al. 1987). AU denotes arbitrary units of β‐galactosidase activity.

Transcriptomics analysis identified genes that were significantly differentially expressed between the suppressor s1 and parental *∆fabH* strains (Figure S1). Among these, *madA* and *dtd* were upregulated > 20‐fold in the suppressor s1 (Figure 2A) relative to the parental *∆fabH* strain (Figure S1, Figure S2). The *E. coli* Regulon Database reports that *yihY* is the second gene in a *yihX*, *yihY*, *dtd* and *madA* operon transcribed from the *yihX* promoter (Figure 1D). The *yihX* and *dtd* genes, respectively, encode a phosphatase and an aminoacyl‐tRNA editing enzyme. The *yihY* gene function is unknown, although it encodes a nonessential inner membrane protein (Daley et al. 2005; Baba et al. 2006). Although now corrected (Ezraty et al. 2005), *yihY* was reported to encode a ribonuclease. Despite their diverse functions, translation of the *yihX‐madA* operon transcript appears tightly coupled because all four open reading frames overlap.

Transcriptional fusions to *lacZ* (with glucose catabolite repression of the chromosomal *lac* operon) confirmed that the s1 mutation had no effect on the *yihX* promoter (Figure 2B), consistent with the transcriptomic data (Figure 2A). The altered *yihY* sequence conferred an ~30‐fold increase in transcription of the downstream *dtd* and *madA* genes, whereas the wild‐type *yihY* sequence had only basal activity (Figure 2C–E). Thus, the suppressor mutation created a *de novo* promoter that drove increased expression of *dtd* and *madA* (Figure 2E).

The second SNP of strain s1, a T → C transition allele in *yihT*, was tested by plasmid expression of both the wild type and mutant alleles in the Δ*fabH* strain. Both plasmids failed to restore vancomycin resistance (Figure S3), indicating that the *yihT* mutation had no role in *∆fabH* suppression.

### 2.3 Mapping the Novel Promoter Within the *yihY* Gene

**FIGURE 3:**
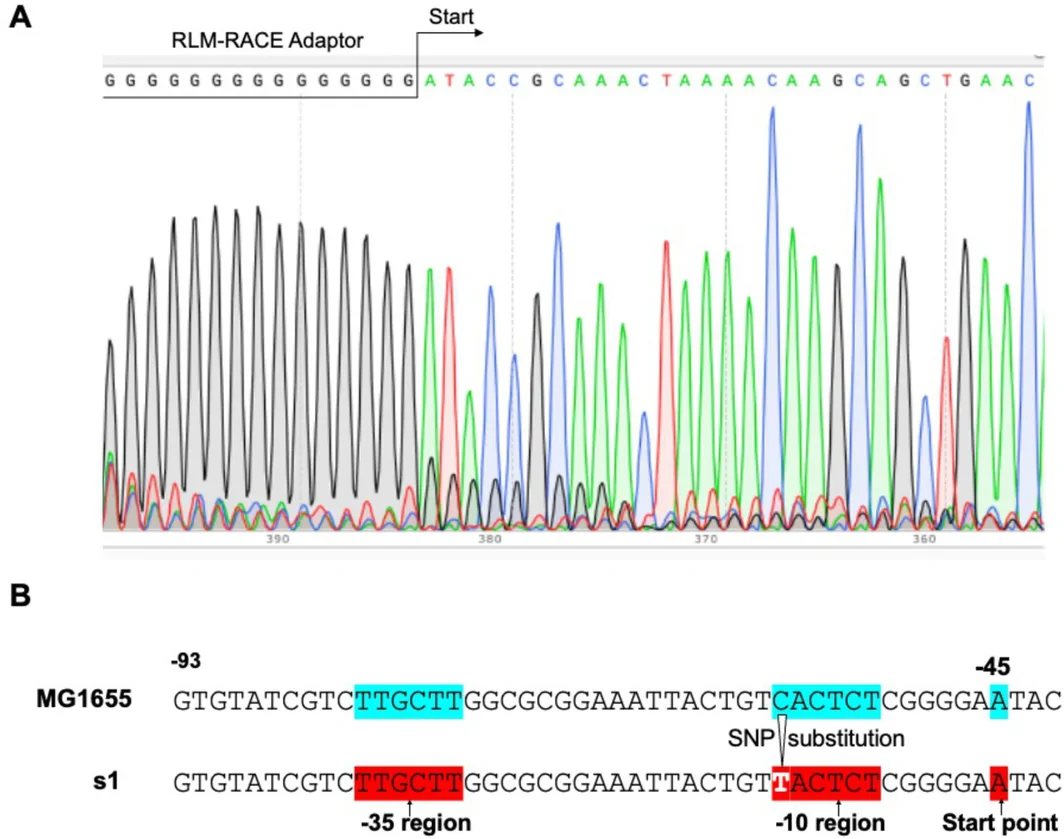
*De novo* promoter formation increases *dtd–madA* transcription in the s1 suppressor strain. (A) Mapping of the transcriptional start site by 5′‐RACE. Representative sequencing chromatogram from analysis of the s1 strain is shown with the mapped transcription start site immediately downstream of the RACE adaptor sequence. The sharp transition from adaptor to genomic sequence defines a discrete transcription initiation site. (B) Promoter architecture underlying suppressor activation. Sequence alignment of MG1655 and the s1 suppressor reveals a C → T transition that generates a functional −10 element (highlighted in red) in the s1 strain. The position of the mapped transcription start site (+1) is indicated. Predicted *σ* ^70^ promoter elements (−35 and −10 regions) are shown, demonstrating that the mutation creates promoter architecture consistent with *σ* ^70^ recognition and appropriate spacing between elements, supporting formation of a functional de novo promoter. The numbers above the sequences give the nucleotide positions relative to *E. coli dtd* gene ATG initiation codon.

The transcription initiation site was determined by 5′‐RACE in parallel with analysis of the *yihY* gene regions of the MG1655 and *∆fabH* strains harvested at mid‐exponential phase. The transcription initiation site of suppressor s1 strain mapped to a site immediately downstream of the promoter created by the C → T transition mutation (Figure 3A). Sequence analysis indicated that the C → T transition generated a near‐canonical *σ* ^70^ promoter by creating a properly positioned −10 element upstream of the transcription start site, 17 bp downstream of a high quality −35 element (Figure 3B) (LaFleur et al. 2022).

### 2.4 MadA Is Responsible for Bypass of *fabH* Deletion and Vancomycin Resistance in Strain s1

The above data demonstrated formation of a new promoter in strain s1 that greatly increased expression of the *dtd‐madA* region (Figures 2 and 3). Expression plasmids encoding either *dtd* or *madA* were constructed and transformed into the *∆fabH* strain. As expected, overexpression of MadA but not Dtd in the *∆fabH* strain resulted in recovery of vancomycin resistance (Figure S4A). Expression of the *Pseudomonas stutzeri* MadB (Figure S5) in the *∆fabH* strain also resulted in vancomycin resistance (Figure S4B) and restored wild‐type levels of *de novo* fatty acid synthesis (Figure S4C). MadB is essentially the C‐terminal malonyl‐ACP decarboxylase domain of MadA (Whaley et al. 2021). The *P. stutzeri* MadB is 83% identical to the *P. putida* MadB (McNaught et al. 2023). The MadA N‐terminal domain affects cyclic‐AMP metabolism (Ciemniecki et al. 2026) and thus the MadB data indicate that the MadA N‐domain has no role in *∆fabH* suppression. High levels of MadA expression were toxic to growth and inhibit fatty acid synthesis (Figures S6 and S7), arguing that the s1 suppressor promoter had to be sufficiently strong to bypass loss of FabH but not so strong to become toxic.

The increased MadA expression of strain s1 restored two other indications of outer membrane function to the *∆fabH* strain: resistance to sodium dodecyl sulfate and swimming motility (Figure S8).

### 2.5 The Function of Malonyl‐ACP Decarboxylation Is to Prime Fatty Acid Synthesis

**FIGURE 4:**
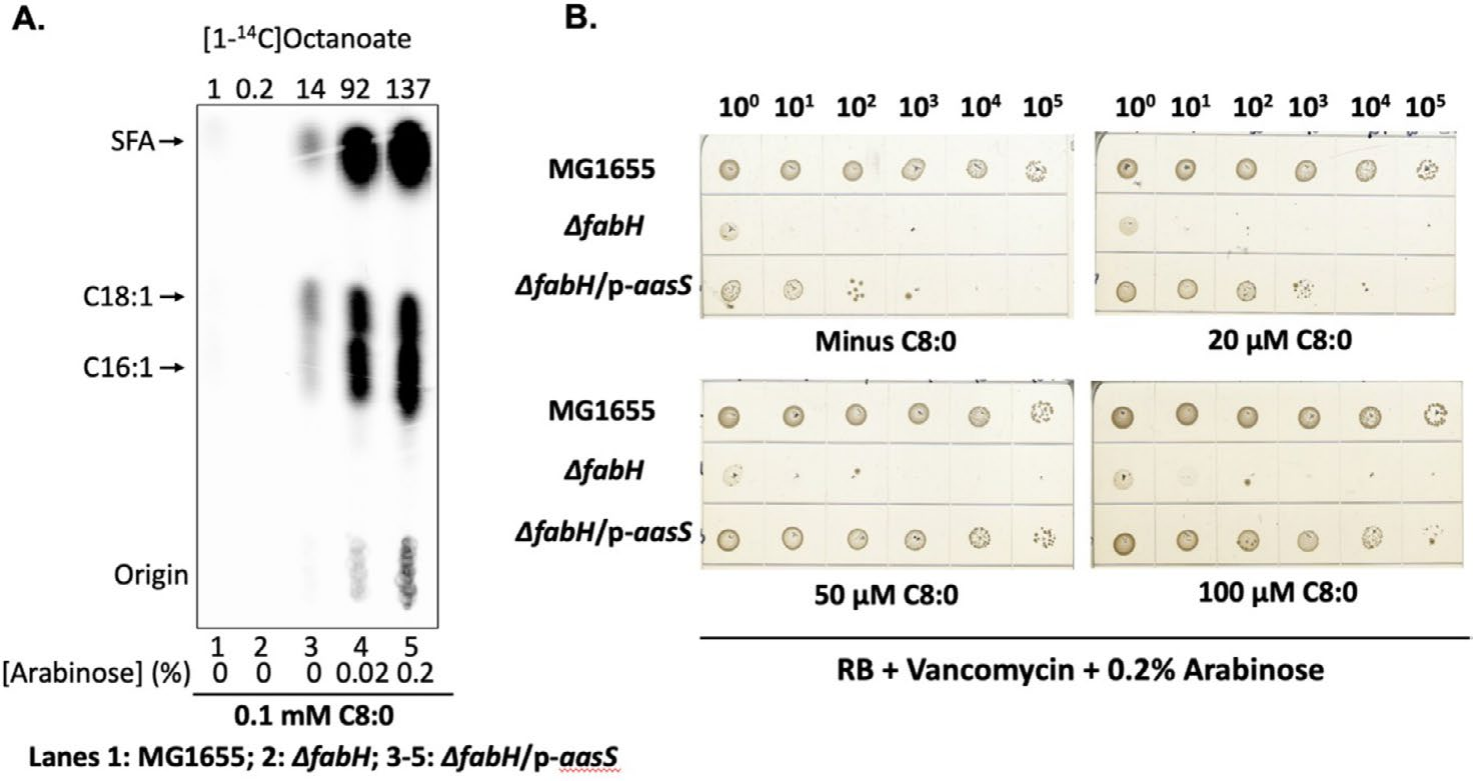
Synthesis of an octanoyl‐ACP primer by *Vibrio harveyi* acyl‐ACP synthase (AasS) restores wild type vancomycin resistance and growth to the *∆fabH* strain. (A) Dependence on AasS induction of [1‐^14^C]octanoate incorporation into phospholipid acyl chains. (B) Restoration of wild type vancomycin resistance requires both AasS induction and octanoate supplementation (the numbers above each figure panel indicate the dilution factor from the overnight cultures). Note that the trace growth of the *∆fabH/p‐aasS* strain can be attributed to the small cytosolic octanoate pool present in *E. coli* (Hermes and Cronan 2009).

To provide a further in vivo test that MadA expression provides an alternate initiation primer and vancomycin resistance, we provided an alternate ACP primer. A *V. harveyi* AasS acyl‐ACP synthetase plasmid (Jiang et al. 2010) was transformed into the *∆fabH* strain with octanoate (C8:0) supplementation to give octanoyl‐ACP, a precursor of both unsaturated and saturated acyl chains (Cronan 2024). The AasS‐octanoate bypass restored vancomycin resistance but only when both AasS expression and octanoate supplementation were present (Figure 4).

## 3 Discussion

FabH was considered essential in *E. coli* because it uniquely initiated fatty acid synthesis (Tsay et al. 1992; Cronan Jr. and Rock 2008). However, MadA showed FabH was not unique (Sanyal et al. 2019; Whaley et al. 2021). MadA decarboxylates malonyl‐ACP to acetyl‐ACP to bypass loss of FabH (Whaley et al. 2021). Wild type MadA provides only limited fatty acid flux. *E. coli* K‐12 studies showed that Δ*fabH* cells are markedly smaller, indicating that fatty acid availability constrains membrane biogenesis and thereby cell size (Yao et al. 2012). Our finding that fatty acid synthesis is reduced to ~30% of wild‐type levels fits well (Figure 1C) with Δ*fabH* cells being ~30% of the wild‐type size (Yao et al. 2012; Whaley et al. 2021). This supports the lipid‐centric framework of Levin and co‐workers (Vadia et al. 2017) in which that fatty acid flux scales closely with envelope expansion capacity. However, our prior work in extra‐intestinal pathogenic *E. coli* (ExPEC) strains presents an important exception (Hong et al. 2022). Despite severe fatty acid limitation, both CFT073 and EC958 Δ*fabH* cells retain near wild‐type size, albeit with slower division, suggesting that these ExPEC strains trade growth rate for maintenance of cell size (Hong et al. 2022).

FabH has long been pursued as an antimicrobial target (Verma et al. 2025) which reflects the common assumption in antibacterial discovery that disruption of a core biosynthetic step will be difficult for bacteria to bypass. The s1 suppressor demonstrates upregulation of *madA* to restore wild‐type growth to the Δ*fabH* strain showing that bypass of loss of a major biosynthetic step can arise through a minimal *cis*‐regulatory change. The s1 mutation in *yihY* converted a sequence into a *σ* ^70^–10 element. The newly introduced T resulted in a TACTCT −10 element that contains both bases (the TA and 3′T) that are key in interactions with *σ* ^70^ (Feklistov and Darst 2011; Sevostyanova et al. 2007). Moreover, free‐energy modeling predicted reduced local duplex stability at this site (Figure S9).

Excessive *madA* expression inhibited phospholipid acyl chain synthesis in wild‐type *E. coli* (Figures S7 and S8). To approximate the level of MadA expression causing toxicity, we expressed MadA from two different promoters in strain MG1655. The weaker arabinose promoter gave a ~3‐fold decrease in phospholipid acyl chain synthesis but only a modest decrease in growth (Figure S7), whereas the more powerful T5 promoter almost completely blocked both growth and acyl chain synthesis (Figure S8). Elevated MadA activity would decrease the malonyl‐ACP pool needed for acyl chain elongation. Expression of an enzyme (BioC) that methylates the free carboxyl of malonyl‐ACP is similarly toxic (Lin and Cronan 2012). Avoidance of toxicity may explain why *madA* transcription requires alarmone activation.

Inhibition of core metabolic pathways may be especially vulnerable to evolutionary bypass when latent compensatory functions already exist that need only transcriptional activation. Indeed, *de novo* promoter formation is highly accessible in *E. coli* . Experimental evolution has shown that ~60% of forty ~100 bp random sequences are only a single base change away from promoter activity whereas ~10% are already active (Yona et al. 2018). Second, the *E. coli* K‐12 genome retains ~200 unannotated putative coding sequences (Goodall et al. 2018). These findings suggest that inhibition of core metabolic pathways is more vulnerable to evolutionary bypass than generally assumed.

## 4 Materials and Methods

### 4.1 Materials

Sigma‐Aldrich and New England Biolabs were the sources of most reagents. Sodium[1‐^14^C] acetate (57.0 mCi/mmol) and sodium[1‐^14^C] octanoate (55.0 mCi/mmol) were supplied by Moravek and American Radiolabeled Chemicals, respectively.

### 4.2 Bacterial Strains, Plasmids and Growth Media

Bacterial strains and plasmids are listed in Table S1 and primers in Table S2. Cultures were incubated at 37°C or 30°C in LB medium (tryptone, 10 g/L; yeast extract, 5 g/L; NaCl, 10 g/L) or RB (LB lacking yeast extract). Antibiotics were added at (in μg/L) sodium ampicillin, 100; chloramphenicol, 30; vancomycin, 50. Growth was determined by OD_600_. with a Bioscreen C.

### 4.3 Genome Sequencing

Genomic DNA using the Qiagen DNeasy blood and tissue kit. Whole genome sequencing was performed by BGI (Hong Kong) using DNBseq platforms generating > 800 bp read data. Sequencing data were aligned to the *E. coli* MG1655 reference genome.

### 4.4 Construction of Expression Plasmids

The plasmids were constructed as described in the legends of the relevant Supporting Information: figures.

### 4.5 Analysis of ^14^C Labeled Phospholipid Acyl Chains

*De novo* synthesis was assayed as described previously (Zou et al. 2022). Cultures (5 mL) from dilution of overnight cultures into fresh medium at OD_600_ 0.05 and adding 1 mCi/L sodium [1‐^14^C]acetate was followed by incubation for 3 h at 37°C. The same protocol except that 0.1 mM cold octanoic acid and 0.1 mCi/L sodium[1‐^14^C]octanoate was used. The [^14^C]‐labeled fatty acid methyl esters were synthesized and analyzed as given (Zou et al. 2022).

### 4.6 LacZ Transcriptional Fusion Constructions and β‐Galactosidase Assay

The effects of the s1 suppressor mutation *yihX* promoter output were tested. A 500 bp upstream region including the first 36 bp of *yihX* (−500 to +36 relative to the *yihX* start codon) was amplified from MG1655 DNA using primers P‐*yihX* F and 5 ‐*yihX* R. Similarly, the *lacZ* coding sequence was obtained using primers *lacZ* F and *lacZ* R. These fragments were inserted into pHSG575 to give the *yihX–lacZ* fusion plasmid. Similar manipulations gave the *dtd‐lacZ* fusion. β‐Galactosidase was assayed in triplicate (Bi et al. 2014; Zou et al. 2022). LB containing glucose was used to repress expression of chromosomal *lacZ*.

### 4.7 Mapping of the *yihY* Transcription Initiation Site

The transcription initiation site was determined using the Sangon Biotech 5′‐RACE Kit Briefly, *E. coli* MG1655, ∆*fabH*, and its suppressor isolate s1 were cultured in LB medium at 37°C to OD_600_ of 0.6. The cells were harvested and total RNA extracted using the Tiangen RNA extraction kit. The concentration and purity of the RNA were by A260/A280 RNA integrity was confirmed by gel demonstration of discrete 23S and 16S ribosomal RNA bands. A specific reverse transcription primer RT1 for *dtd‐madA* was utilized to synthesize cDNA from the mRNA template by the Reverse Transcriptase Mix (RNase H‐), followed by addition of a homopolymeric (dC) tail of 10–15 residues to the 3′ end of the cDNA by terminal transferase (TdT) to allow pairing with the 5′ adaptor primer (GCTGTCAACGATACGCTACGTAACGGCATGACAGTGGGGGGGGGGGGG) containing an oligonucleotide anchor sequence. The first‐round PCR amplification was performed using this adaptor primer and a specific outer primer R1of the *dtd‐madA* region with the single‐stranded cDNA as the template. A second (nested) PCR was performed using the 5′ RACE outer primer (GCTGTCAACGATACGCTACGTAAC), which contained a partial adapter sequence and R2 another specific inner primer of the *dtd‐madA* cluster to amplify the 5′‐terminal cDNA fragment of the *dtd* gene. The transcription initiation site wase determined by sequencing of the final PCR products by Sangon Biotech.

## Author Contributions

**Qiaoqiao Guo:** investigation, methodology, validation, writing – review and editing, writing – original draft. **Jilong Qin:** investigation, formal analysis. **Yaoqin Hong:** conceptualization, investigation, methodology, validation, writing – original draft, writing – review and editing. **Qi Zou:** investigation, methodology, validation, writing – original draft, writing – review and editing. **John E. Cronan:** conceptualization, investigation, funding acquisition, writing – original draft, methodology, validation, writing – review and editing, project administration, supervision. **Makrina Totsika:** investigation, project administration, funding acquisition.

## Funding

This work was supported by the National Institute of Allergy and Infectious Diseases, AI15650. Queensland University of Technology, 2017HIG0119. James Cook University.

## Ethics Statement

The authors have nothing to report.

## Conflicts of Interest

Makrina Totsika is an employee of the GSK group of companies. This research was conducted independently of any commercial interests. The remaining authors declare no conflicts of interest.

## Supporting information


**Figure S1:** Gene ontology (GO) enrichment analysis was performed with genes that exhibit a differential expression log2 ratio ≥ 3.0 using ShinyGO (Ge et al. 2019).
**Figure S2:** Differentially expressed genes with ultra‐high absolute confidence between three replicates of ∆*fabH* and suppressor s1. RNA was extracted and purified using the Qiagen RNeasy kits for RNA purification, in accordance with manufacturer's protocol. Transcriptomic sequencing was performed by BGI (Hong Kong) using DNBse platforms. Sequencing data were mapped to the *E. coli* MG1655 reference genome. (A) The differentially expressed genes between ∆*fabH* and suppressor s1 (hits with absolute confidence at −1000 or 1000 are highlighted by yellow transparent strips at the top of the volcano plot). Principal component analysis showed clear separation of conditions along PC1 (98.6% variance), indicating the primary source of transcriptional variation is driven by the experimental condition. Replicates clustered tightly within each group with only minor variation observed along PC2 (0.52%), suggesting limited technical or biological noise. (B) Sankey plot illustrating the identity of genes highlighted in the volcano plot and categorized into relevant pathways (genes in bold were upregulated and vice versa). The *madA* gene is highlighted with an orange oval. Many of the transcriptional changes can be attributed to restoration of a functional outer membrane in the s1 suppressor strain. The Sankey plot was visualized with Omicshare tools (Mu et al. 2024).
**Figure S3:** Plasmid expression of the wild‐type and mutant *yihT* failed to reverse vancomycin sensitivity of the ∆*fabH* strain. The C‐T transition mutant allele of *yihT* is denoted as yihT*. Overnight cultures were adjusted to OD_600_ and serially diluted 10‐fold, with 5 μL spotted onto LB agar containing 50 μM vancomycin and grown overnight at 37°C.
**Figure S4:** Overexpression of MadA or expression of MadB restored growth in the presence of vancomycin and wild type fatty acid synthesis. (A) Vancomycin resistance of MG1655 ∆*fabH* was restored upon overexpression of MadA or the *P. stutzeri* MadB homologue. (B) Expression of the *P. stutzeri* MadB restored vancomycin resistance to the *∆fabH* strain. In panels A and B, the numbers above each figure panel indicate the dilution factor from the overnight cultures. (C) [1‐^14^C]acetate incorporation to measure *de nov*o fatty acid biosynthesis in the *∆fabH* strain with either overexpression of MadA or expression of MadB. In panel C the numbers above the lanes give the phospholipid unsaturation and the radioactive label incorporation values relative to the value (100) for the wild type MG1655 strain. The *dtd* and *madA* expression plasmids for *E. coli dtd* and *madA* genes, were constructed from MG1655 strain genomic DNA using primer sets *dtd* NdeI F and *dtd* HindIII R, and *madA* NdeI F and *madA* HindIII R, respectively, inserted into the NdeI and HindIII sites of vector pBAD24M. The *dtd‐madA* fragment was amplified using primer set *dtd* NdeI F and *madA* HindIII R and cloned as above. The *yihT* coding region was amplified from the genomic DNAs from MG1655 or the s1 suppressor strains by primer set *yihT* F1 and *yihT* R1 and then ligated with linearized vector pHSG575 by Gibson assembly.
**Figure S5:** The *Pseudomonas stutzeri* ATCC 17588 = LMG 11199 *madB* gene and MadB protein (UniProt A0A0H3YTL2_STUST). Note that *P. stuteri* is now called Stutzerimonas stutzeri in some data bases.
**Figure S6:** Effects of p*araBAD* promoter driven MadA overproduction on fatty acid synthesis and growth of strain MG1655 (denoted as WT). (A) [1‐^14^C]acetate incorporation into phospholipid acyl chains as referenced in Materials and Methods. The cultures were started at an OD_600_ of 0.05 for 1 h followed by addition of 1 mCi/L [1‐^14^C]acetate with or without supplementation with 0.2% arabinose for another 3 h. (B) Growth of MG1655 and MG1655 carrying the p‐*madA* plasmid in the presence or absence of 0.2% arabinose. The medium was RB, a relatively poor medium such that arabinose consumption stimulated growth. Note that growth of the p‐*madA* culture overexpressing MadA that contained arabinose ceased growth after about 4 h. The expression plasmid the *madA* genes, was amplified from *E. coli* MG1655 strain genomic DNA using primer sets *madA* NdeI F and *madA* HindIII R, respectively, and then inserted into the NdeI and HindIII sites of vector pBAD24M.
**Figure S7:** Effects of pT5 promoter driven MadA overproduction on growth of strain MG1655 (denoted as WT) and fatty acyl chain synthesis. (A) Growth on LB plate (essentially the same result was given on RB plates). (B) Growth in RB medium, IPTG 1 mM was added as given. (C) [^14^C]acetate incorporation into membrane phospholipid acyl chains. The cultures were started at 37°C in RB medium at an OD_600_ of 0.05 and incubated to log phase (OD_600_ of about 0.3–0.4) followed by addition of 1 mCi/L of [1‐^14^C]acetate with or without supplementation with 1 mM IPTG for another 3 h. The radiolabeled fatty acyl chains were analyzed as described in Materials and Methods. For *madA* expression from the phage T5 promoter, the *madA* coding region was applified from MG1655 DNA using primer set Ec madA F2 and Ec madA R2 and then ligated to vector pQE2 vector.
**Figure S8:** Other properties of the s1 and *∆fabH*/p‐*aasS* strains. The top and middle panels show relief of the sodium dodecyl sulfate (SDS) sensitivity of *∆fabH* strains by the s1 mutation and the octanoate‐dependent production of acyl‐ACP synthase, respectively. The bottom panel shows the s1 mutation restores swimming motility to the *∆fabH* strain assayed in 0.3% LB agar after spotting 10 μL of each culture and incubation for ~40 h. The strains were: top left MG1655, top right *∆fabH* and lower right, s1 (Bp7 was an early name for strain s1).
**Figure S9:** Predicted changes in promoter free energy (ΔG_total_) associated with the s1 suppressor mutation. Predictions were generated using a model‐based promoter calculator that integrates sequence‐dependent contributions to transcription initiation, including *σ* ^70^–DNA interactions and local DNA energetics (LaFleur et al. 2022). The s1 suppressor exhibits a local decrease in ΔG_total_ relative to Δ*fabH*, consistent with increased promoter favorability at the mutated site. Transcriptional prediction and ∆*G* energy/position‐weight modellings were performed as previously described (LaFleur et al. 2022).
**Table S1:** Strains and Plasmids used in this work.
**Table S2:** Sequences of the PCR primers used.

## Data Availability

The raw sequence data reported in this paper have been deposited in the Genome Sequence Archive (Zhang et al. 2025) in National Genomics Data Center (Members and Partners 2026), China National Center for Bioinformation/Beijing Institute of Genomics, Chinese Academy of Sciences (GSA: CRA043388) that are publicly accessible at https://ngdc.cncb.ac.cn/gsa. Additional data that support the findings of this study are available on request from the corresponding author.
