# Supplementary material for: A Single‐Nucleotide Substitution Generates a de Novo Promoter That Activates a Latent Metabolic Bypass in Escherichia coli
Source: Mol Microbiol. 2026 Jun 26;126(3):244–51. doi: 10.1111/mmi.70092 (PMC13555641; doi:10.1111/mmi.70092)
Supplement: Supplementary file 1 [file MMI-126-244-s001.docx]

**Supplemental Material**

**A single-nucleotide substitution generates a *de novo* promoter that activates a latent metabolic bypass in *Escherichia coli.***

Qiaoqiao Guo, Qi Zou, Jilong Qin, Makrina Totsika, Yaoqin Hong, John E. Cronan

Fig. S1. Gene ontology (GO) enrichment analysis was performed with genes that exhibit a differential expression log2 ratio ≥ 3.0 using ShinyGO (Ge *et al.*, 2019).

Fig. S2. Differentially expressed genes with ultra-high absolute confidence between three replicates of ∆*fabH* and suppressor s1. RNA was extracted and purified using the Qiagen RNeasy kits for RNA purification, in accordance with manufacturer’s protocol. Transcriptomic sequencing was performed by BGI (Hong Kong) using DNBse platforms. Sequencing data were mapped to the *E. coli* MG1655 reference genome. **A**. The differentially expressed genes between ∆*fabH* and suppressor s1 (hits with absolute confidence at -1000 or 1000 are highlighted by yellow transparent strips at the top of the volcano plot). Principal component analysis showed clear separation of conditions along PC1 (98.6% variance), indicating the primary source of transcriptional variation is driven by the experimental condition. Replicates clustered tightly within each group with only minor variation observed along PC2 (0.52%), suggesting limited technical or biological noise. **B**. Sankey plot illustrating the identity of genes highlighted in the volcano plot and categorized into relevant pathways (genes in bold were upregulated and vice versa). The *madA* gene is highlighted with an orange oval. Many of the transcriptional changes can be attributed to restoration of a functional outer membrane in the s1 suppressor strain. The Sankey plot was visualized with Omicshare tools (Mu *et al.*, 2024).

Fig. S3. Plasmid expression of the wild-type and mutant *yihT* failed to reverse vancomycin sensitivity of the ∆*fabH* strain. The C-T transition mutant allele of *yihT* is denoted as *yihT*.* Overnight cultures were adjusted to OD_600_ and serially diluted 10-fold, with 5 µL spotted onto LB agar containing 50 µM vancomycin and grown overnight at 37 ℃.

Fig. S4. Overexpression of MadA or expression of MadB restored growth in the presence of vancomycin and wild type fatty acid synthesis. (**A**) Vancomycin resistance of MG1655 ∆*fabH* was restored upon overexpression of MadA or the *P. stutzeri* MadB homologue. (**B**) Expression of the *P. stutzeri* MadB restored vancomycin resistance to the *∆fabH* strain. In panels A and B, the numbers above each figure panel indicate the dilution factor from the overnight cultures. (**C**) [1-^14^C]acetate incorporation to measure *de nov*o fatty acid biosynthesis in the *∆fabH* strain with either overexpression of MadA or expression of MadB. In panel **C** the numbers above the lanes give the phospholipid unsaturation and the radioactive label incorporation values relative to the value (100) for the wild type MG1655 strain. The *dtd* and *madA* expression plasmids for *E. coli dtd* and *madA* genes, were constructed from MG1655 strain genomic DNA using primer sets *dtd* NdeI F and *dtd* HindIII R, and *madA* NdeI F and *madA* HindIII R, respectively, inserted into the NdeI and HindIII sites of vector pBAD24M.The *dtd-madA* fragment was amplified using primer set *dtd* NdeI F and *madA* HindIII R and clone as above. The *yihT* coding region was amplified from the genomic DNAs from MG1655 or the s1 suppressor strains by primer set *yihT* F1 and *yihT* R1 and then ligated with linearized vector pHSG575 by Gibson assembly.

Fig S5. The *Pseudomonas stutzeri* ATCC 17588 = LMG 11199 *madB* gene and MadB protein (UniProt A0A0H3YTL2_STUST). Note that *P. stuteri* is now called [*Stutzerimonas stutzeri*](https://www.uniprot.org/taxonomy/316) in some data bases.

***madB***

ATGAGCCGCGATAGCCGCTACCTGGAAGCCATCCTTCACCACGACATTCCGCTGACCCGCTCGCTTGGGTTGCGTGTCGAGCGTGGGAAAACCACGAGCTGCGCCTGCAGGTCCCGCTGCAGCCGAACATCAACCACAAGAGCAGCATGTTCGGCGGCAGCCTCTACTGCGCCGCCGTACTGGCAGGCTGGGGCTGGCTGCATCTGCGCCTGCGCGAAGCCGGCATCGAAGACGGCCACATCGTCATCCACGAAGGCAAGATCGAATACCCGCTGCCGGTGGTCGACGACGCCATGGCCATCTGTTCGGCACCGAGCCCCGAGGCCTGGGAACGTTTCGAGTCGATCTACCGCCGGCGCGGGCGCTCGCGCCTCGAGCTGCACAGCCGCATCCTCGCCTCCGACGGTCGCGACGCCGTGCGCTTCACCGGGCAGTTCGTGCTGCACAAGTAA

**MadB**

MSRDSRYLEAILHHDIPLTRSLGLRVEQWENHELRLQVPLQPNINHKSSMFGGSLYCAAVLAGWGWLHLRLREAGIEDGHIVIHEGKIEYPLPVVDDAMAICSAPSPEAWERFESIYRRRGRSRLELHSRILASDGRDAVRFTGQFVLHK

Fig. S6. Effects of p*araBAD* promoter driven MadA overproduction on fatty acid synthesis and growth of strain MG1655 (denoted as WT). (**A**) [1-^14^C]acetate incorporation into phospholipid acyl chains as referenced in Materials and Methods. The cultures were started at an OD_600_ of 0.05 for 1 h followed by addition of 1 mCi/L [1-^14^C]acetate with or without supplementation with 0.2% arabinose for another 3 h. (**B)** Growth of MG1655 and MG1655 carrying the p-*madA* plasmid in the presence or absence of 0.2% arabinose. The medium was RB, a relatively poor medium such that arabinose consumption stimulated growth. Note that growth of the p-*madA* culture overexpressing MadA that contained arabinose ceased growth after about 4 h. The expression plasmid the *madA* genes, was amplified from *E. coli* MG1655 strain genomic DNA using primer sets *madA* NdeI F and *madA* HindIII R, respectively, and then inserted into the NdeI and HindIII sites of vector pBAD24M.

Fig. S7. Effects of pT5 promoter driven MadA overproduction on growth of strain MG1655 (denoted as WT) and fatty acyl chain synthesis. **A**. Growth on LB plate (essentially the same result was given on RB plates). **B.** Growth in RB medium, IPTG 1 mM was added as given. **C.** [^14^C]acetate incorporation into membrane phospholipid acyl chains. The cultures were started at 37℃ in RB medium at an OD_600_ of 0.05 and incubated to log phase (OD_600_ of about 0.3 to 0.4) followed by addition of 1 mCi/L of [1-^14^C]acetate with or without supplementation with 1 mM IPTG for another 3 h. The radiolabeled fatty acyl chains were analyzed as described in Materials and Methods. For *madA* expression from the phage T5 promoter, the *madA* coding region was applified from MG1655 DNA using primer set Ec madA F2 and Ec madA R2 and then ligated to vector pQE2 vector.

Fig. S8. Other properties of the s1 and *∆fabH*/p-*aasS* strains. The top and middle panels show relief of the sodium dodecyl sulfate (SDS) sensitivity of *∆fabH* strains by the s1 mutation and the octanoate-dependent production of acyl-ACP synthase, respectively. The bottom panel shows the s1 mutation restores swimming motility to the *∆fabH* strain assayed in 0.3% LB agar after spotting 10 μl of each culture and incubation for ~40h. The strains were: top left MG1655, top right *∆fabH* and lower right, s1 (Bp7 was an early name for strain s1).

Fig. S9. Predicted changes in promoter free energy (ΔG_total_) associated with the s1 suppressor mutation. Predictions were generated using a model-based promoter calculator that integrates sequence-dependent contributions to transcription initiation, including σ⁷⁰–DNA interactions and local DNA energetics (LaFleur *et al.*, 2022). The s1 suppressor exhibits a local decrease in ΔG_total_ relative to Δ*fabH*, consistent with increased promoter favorability at the mutated site. Transcriptional prediction and ∆*G* energy/position-weight modellings were performed as previously described (LaFleur *et al.*, 2022).

**Table S1. Strains and Plasmids used in this work**

| **Strain or plasmid** | | **Relevant characteristic(s)** | **Source/Reference** |
| --- | --- | --- | --- |
| **Strains** | |  |  |
| MG1655 | *Escherichia coli* K-12 wild type strain, F^-^ l^-^ *ilvG rfb-50 rph-1* | | Coli Genetic Stock Center |
| MT1801 | MG1655 *∆fabH* | | (Hong *et al.*,  2022) |
| MT2871 | MG1655 *∆fabH* bypass strain (s1) | | This work |
| GQQ111 | MG1655 with *lacZ* expression from *yihX* promoter in pHSG575 | | This work |
| GQQ112 | MG1655 *∆fabH* with *lacZ* expression from *yihX* promoter in pHSG575 | | This work |
| GQQ113 | MG1655 *∆fabH* bypass strain with *lacZ* expression from *yihX* promote*r* in pHSG575 | | This work |
| GQQ120 | MG1655 *∆fabH* with *yihT* expression in pSRK | | This work |
| GQQ121 | MG1655 *∆fabH*: with *yihT* (K109R) expression in pSRK | | This work |
| GQQ124 | MG1655 *∆fabH* with *yihT* expression in pHSG575 | | This work |
| GQQ125 | MG1655 *∆fabH* with *yihT* (K109R) expression in pHSG575 | | This work |
| GQQ126 | MG1655 *∆fabH* with *yihT* expression in pHSG575 | | This work |
| GQQ127 | MG1655 *∆fabH* with *yihT* (K109R) expression in pHSG575 | | This work |
| GQQ155 | MG1655 with *lacZ* expression from s1 promoter in pHSG575 | | This work |
| GQQ157*^a^* | MG1655 with *lacZ* expression from wild type *yihY* sequence in pHSG575 | | This work |
| GQQ158 | MG1655 *∆fabH* bypass strain with *lacZ* expression from s1 promoter in pHSG575 | | This work |
| GQQ159*^a^* | MG1655 *∆fabH bypass strain* with *lacZ* expression from wild type *yihY* sequence in pHSG575 | | This work |
| GQQ160 | MG1655 *∆fabH* with *lacZ* expression from s1 promoter in pHSG575 | | This work |
| GQQ161*^a^* | MG1655 *∆fabH* with *lacZ* expression from wild type *yihY* sequence in pHSG575 | | This work |
| GQQ162 | MG1655 *∆fabH* with Vh*aasS* expression in pBAD33 | | This work |
| QZ601 | MG1655 *∆fabH* with *dtd* expression in pBAD24M | | This work |
| QZ602 | MG1655 *∆fabH* with *madA* expression in pBAD24M | | This work |
| QZ603 | MG1655 *∆fabH* with *dtd-madA* co-expression in pBAD24M | | This work |
| QZ650 | MG1655 *∆fabH* with *fabH* expression in pBAD24M | | This work |
| QZ651 | MG1655 *∆fabH* with Psv*madB* expression in pBAD24M | | This work |
| QZ38 | MG1655 with *madA* expression in pBAD24M | | This work |
| QZ64 | MG1655 with *madA* expression in pQE2 | |  |
| **Plasmids** |  | |  |
| pBAD24M | Amp^r^; NcoI site of expression vector pBAD24 changed to an NdeI site | | (Guzman *et al.*,  1995) |
| pHSG575 | Low copy number vector (pSC101 ori) Cm^r^ | | (Takeshita *et al.*,  1987) |
| pGQQ109 | *lacZ* expression from *yihX* promoter in pHSG575 | | This work |
| pGQQ122 | *yihT* in pHSG575 | | This work |
| pGQQ123 | *yihT* (K109R) in pHSG575 | | This work |
| pGQQ152 | *lacZ* expression from wild type *yihY* sequence in pHSG575 | | This work |
| pGQQ156 | *lacZ* expression from s1 promoter in pHSG575 | | This work |
| pYFJ86 | Vh*aasS* expression in pBAD33 | | (Hu *et al*., 2018) |
| pQZ598 | *dtd* expression in pBAD24M | | This work |
| pQZ599 | *madA* expression in pBAD24M | | This work |
| pQZ600 | *dtd-madA* co-expression in pBAD24M | | This work |
| pQZ61 | *madA* expression in pQE2 | | This work |
| pYY606 | *fabH* expression in pBAD24M | | Lab Store |
| pYY682 | Psv*madB* expression in pBAD24M | | (Yin, *et al*., in preparation) |

*^a^*In strains GQQ157, 159 and 161, the DNA segment of the wild type *yhiY* sequence was the same as that containing the promoter of the s1 suppressor strain.

**Table S2. Sequences of the PCR primers used**

| **Primer name** | **Primer sequence (5′-3′)** |
| --- | --- |
| pHSG575-lacZ F | ATGACCATGATTACGGATTCA |
| pHSG575-lacZ R | GCCTGGGGTGCCTAA |
| P-dtd F | GAGTTAGCTCACTCATTAGGCACCCCAGGCTTGAATACCATCTGGCG |
| 5'-dtd R | GACGGCCAGTGAATCCGTAATCATGGTCATGCTGGCACGGGTTAC |
| dtd NdeI F | GGGAATTCCATATGATTGCATTAATTCAACGC |
| dtd HindIII R | CTAGAAGCTTTCATACCTGCAACCAGAA |
| madA NdeI F | GGGAATTCCATATGAGCCAGCTTCCA |
| madA HindIII R | CTAGAAGCTTCTACTCTTCTTCGTTCCC |
| yihT F1 | TAACAATTTCACACAGGAAACAGATGAATAAGTACACCATCAACG |
| yihT R1 | CGTTGTAAAACGACGGCCAGTGAATTCTTAGCGGCGTTTAGCCATCAT |
| pHSG575 F1 | GAATTCACTGGCCGTCGTTTTACAACG |
| pHSG575 R1 | CTGTTTCCTGTGTGAAATTGTTA |
| pHSG575 F2 | GTTGGTCTGGTGTCAAAAATAATAACCGTTTTTATCAGGCTCTGGG |
| pHSG575 R2 | GTGGTAAAGAATATGCGCCACACGGCCTGGGGTGCCTAATGAGTGAG |
| LacZ F | GATTTAGGTAATGTGATTGTCATGACCATGATTACGGATTC |
| LacZ R | CCCAGAGCCTGATAAAAACGGTTATTATTTTTGACACCAGACCAAC |
| P-yihX F | CTCACTCATTAGGCACCCCAGGCCGTGTGGCGCATATTCTTTACCAC |
| P-yihX R | GAATCCGTAATCATGGTCATGACAATCACATTACCTAAATC |
| madA F2 | ACAGAATTCATTAAAGAGGAGAAATTAACTATGAGCCAGCTTCCA |
| madA R2 | ATCTATCAACAGGAGTCCAAGCTCAGCTAACTACTCTTCTTCGTTCCC |
| pQE2 F | TTAGCTGAGCTTGGACT |
| pQE2 R | AGTTAATTTCTCCTCTTTAATGAATT |
